# The Clinical Significance of Schistocytes: A Prospective Evaluation of the International Council for Standardization in Hematology Schistocyte Guidelines

**DOI:** 10.4274/tjh.2016.0359

**Published:** 2017-03-01

**Authors:** Elise Schapkaitz, Michael Halefom Mezgebe

**Affiliations:** 1 University of Witwatersrand Medical School, Department of Molecular Medicine and Hematology, Johannesburg, South Africa

**Keywords:** Schistocyte, thrombotic microangiopathy, Microscopy, ADVIA 2120, Standardization

## Abstract

**Objective:**

The presence of ≥1% schistocytes on a peripheral blood smear (PBS) is an important criterion for the diagnosis of thrombotic microangiopathy (TMA). The reporting of schistocytes has been standardized by the International Council for Standardization in Hematology (ICSH). Despite the availability of guidelines, however, the assessment of schistocytes remains subjective. More recently, the automated fragmented red cell (FRC) parameter has been evaluated. However, local studies are not available.

**Materials and Methods:**

A prospective study was performed at the Charlotte Maxeke Johannesburg Academic Hospital in order to evaluate the ICSH recommendations for schistocyte measurement in 146 PBSs with schistocytes. Schistocytes were evaluated by microscopy and ADVIA 2120 automated hematology analyzers.

**Results:**

Schistocytes were frequently observed in patients with TMA (n=76), infection (n=20), hematologic malignancy (n=10), renal failure (n=5), and hemoglobinopathy (n=15), and in neonates (n=11). Schistocytes were ≥1% in all PBSs with TMA (n=76) with a mean of 3.44±1.84. Schistocytes of ≥1% were also observed in cases of renal failure and hemoglobinopathy, and in neonates. In these conditions, schistocytes were mainly observed in conjunction with moderate red blood cell changes. The agreement between two morphologists gave a correlation coefficient of 0.63 [confidence interval (CI): 0.52-0.75], while the correlation coefficient between the average of the morphologists and the FRC percentage was -1.97 (CI: -1.60 to -2.34). The ADVIA 2120 underestimated the schistocyte count in patients with TMA.

**Conclusion:**

Observer bias can be decreased by implementing the standardized procedures recommended by the ICSH. However, estimation of schistocytes by the ADVIA 2120 analyzer requires further evaluation as a screening tool. A higher threshold for schistocytes in thrombotic thrombocytopenic purpura is recommended to distinguish this hematological emergency from other conditions associated with ≥1% schistocytes.

## Abstract (tr)

**Amaç:**

Periferk kan yaymasında (PKY) ≥%1 şistosit varlığı trombotik mikroanjiopati (TMA) tanısı için önemli bir kriterdir. Şistositlerin raporlanması Hematoloji Standardizasyon Uluslararası Komitesi [International Council for Standardization in Hematology (ICSH)] tarafından standardize edilmiştir. Kılavuzların mevcudiyetine rağmen, şistositlerin değerlendirmesi yine de subjektif kalmaktadır. Son zamanlarda, otomatize fragmente eritrosit (FE) parametresi değerlendirilmektedir. Ne var ki, lokal çalışmalar mevcut değildir.

**Gereç ve Yöntemler:**

ICSH önerilerini değerlendirmek için, Charlotte Maxeke Johannesburg Akademik Hastanesi’nde şistosit saptanan 146 PKY’da şistosit ölçümünü değerlendiren prospektif bir çalışma gerçekleştirildi. Şistositler mikroskop ve ADVIA 2120 otomatize hematoloji analizörü ile değerlendirildi.

**Bulgular:**

Şistositler, TMA (n=76), enfeksiyon (n=20), hematolojik malignite (n=10), renal yetmezlik (n=5) ve hemoglobinopati (n=15) hastalarında ve yenidoğanlarda (n=11) sıklıkla izlendi. Tüm TMA’lı hastaların (n=76) PKY’lerinde şistositler 3,44±1,84 ortalama ile ≥%1 idi. Şistositler ayrıca renal yetmezlik ve hemoglobinopati olguları ve yenidoğanlarda ≥%1 olarak izlendi. Bu durumlarda, şistositler çoğunlukla ılımlı eritrosit değişiklikleri ile ilişkili olarak gözlendi. İki morfolojist arasındaki anlaşma 0,63 [güven aralığı (GA): 0,52-0,75] korelasyon katsayısı verirken, morfolojistlerin ortalaması ve FE yüzdesi arasındaki korelasyon katsayısı -1.97 (GA: -1,60 - -2,34) idi. ADVIA 2120 ile TMA’lı hastalarda şistosit sayısı daha düşük ölçüldü.

**Sonuç:**

ICSH tarafından önerilen standardize prosedürlerin uygulanması ile gözlemci önyargısı azaltılabilir. Ne var ki, tarama aracı olarak ADVIA 2120 analizörü ile şistosit ölçümü daha ileri değerlendirme gerektirmektedir. Trombotik trombositopenik purpurada, şistositlerin ≥%1 olduğu diğer durumlardan bu hematolojik acili ayırt etmek için daha yüksek bir şistosit eşik değeri önerilmektedir.

## INTRODUCTION

Schistocytes are red blood cell (RBC) fragments. The presence of schistocytes on a peripheral blood smear (PBS) according to laboratory policies is a hematological emergency that requires prompt review and investigation for thrombotic microangiopathy (TMA). Schistocytes, however, are not specific to TMA [1]. Fragmentation of RBCs is produced by mechanical damage in the circulation and can also be seen in patients with mechanical heart valves or those receiving dialysis. In addition, schistocytes occur in cytoskeletal RBC abnormalities such as acquired and inherited RBC disorders in association with marked anisopoikilocytosis.

Furthermore, observer bias has been described when identifying and enumerating schistocytes by microscopy [2]. Recently, the identification and diagnostic value of schistocytes was standardized by the International Council for Standardization in Hematology (ICSH) Schistocyte Working Group. According to the ICSH recommendations, the presence of ≥1% schistocytes on a PBS in the absence of other moderate RBC changes is an important criterion for the diagnosis of TMA [3]. Despite the availability of guidelines, laboratory surveys in France indicated that the morphologic identification of schistocytes remained difficult and subjective [4]. More recently, measurement of the automated fragmented red cell (FRC) parameter has been evaluated. Studies have demonstrated that the automated FRC parameter offers advantages such as improved precision, immediate availability, and good agreement with microscopy [5,6,7]. As such, the ICSH Working Group recommended the automated counting of RBC fragments as a useful routine screening tool in the laboratory [3].

A study was performed at the Charlotte Maxeke Johannesburg Academic Hospital (CMJAH) in order to evaluate the ICSH recommendations for schistocyte identification and enumeration in referred PBSs with schistocytes.

## MATERIALS AND METHODS

### Study Design

A laboratory-based prospective study of the PBSs referred for microscopy was performed at the National Health Laboratory Service Hematology Laboratory at the CMJAH, South Africa, from November 2015 to June 2016. One hundred and forty-six PBSs with schistocytes were included. Aged samples were excluded. Clinical information was obtained from the laboratory information system, namely patient characteristics and diagnoses and laboratory investigations including lactate dehydrogenase (LDH) and full blood count (FBC).

### Study Protocol

### Laboratory Methods

Schistocytes were identified on PBSs stained according to the May-Grünwald-Giemsa technique. Schistocytes were defined and counted according to ICSH recommendations [3]. Blinded review of the studied PBSs was independently performed by two competent morphologists (ES and MHM). The schistocyte percentage was estimated by counting 10,000 RBCs at 50x power magnification.

The microscopic schistocyte percentage was compared with the automated FRC percentage measured by ADVIA 2120 hematology analyzers. The automated FRC percentage was determined from measurement of light scatter at two different angles. This corresponded to the refractive index and volume on the platelet scatter plot, which allowed for distinction between platelets and small RBCs. The threshold for the automated FRC parameter was a volume of <30 fL and a refractive index of >1.4 above a threshold of 10,000 events/µL. The percentage of schistocytes was determined from the number of RBCs measured by the analyzer (reference interval for FRC parameter: between 0.2% and 0.3%) [5]. The FBC parameters were measured using ADVIA 2120 hematology analyzers.

### Statistical Analysis

Statistical analysis was performed using the intraclass correlation coefficient (ICCC) as determined by the Bland and Altman method. Statistical comparisons were performed using the parametric paired t-test and nonparametric Wilcoxon matched pairs test for continuous parameters depending upon normality between the TMA and non-TMA groups. Statistical significance was set at a p-value of 0.05 or less.

### Ethics

This study was approved by the Human Research Ethics Committee of the University of the Witwatersrand (M090688).

## RESULTS

The average age of the patients in the study was 26±21 years, with a male-to-female ratio of 1:1.4. Patients were categorized according to diagnosis. Schistocytes were observed in patients with TMA (n=76), infection (n=20), hematologic malignancy (n=10), mechanical heart valves (n=2), renal failure (n=10), hemoglobinopathy (n=15), iron deficiency anemia (n=1), and megaloblastic anemia (n=1) and in neonates (n=11) (Table 1). Patients with TMA had diagnoses such as thrombotic thrombocytopenic purpura (TTP), hemolytic uremic syndrome (HUS), disseminated intravascular coagulopathy (DIC), and hemolysis with elevated liver enzymes and low platelets (HELLP).

The schistocyte counts were normally distributed in the TMA and non-TMA groups with mean (±SD) values of 3.44±1.84% and 1.11±0.83%, respectively (p<0.0001). In addition, the mean values for hemoglobin and platelet count were significantly lower in the TMA group (p<0.049 and p<0.001 respectively). The red cell distribution width (RDW) was significantly higher in the TMA group, whereas there was no difference for LDH (Table 2).

Schistocytes were ≥1% in all PBSs reviewed from patients with TMA (n=76). The mean schistocyte percentages of PBSs from cases of TTP (n=68), HUS (n=1), DIC (n=1), and HELLP (n=5) were 3.51±1.88%, 3.50%, 4.1%, and 2.42±1.6%, respectively. Schistocytes in DIC are frequently observed at lower percentages [8]. In this study, the patient with DIC had concomitant conditions associated with increased schistocytes such as metastatic carcinoma and severe postoperative infection. Schistocytes were observed in the absence of moderate RBC abnormalities in the PBSs reviewed from cases of TMA (with the exception of a case of HUS with moderate crenated cells). Schistocytes of ≥1% were also observed in other conditions, namely renal failure and hemoglobinopathy, and in neonates. In contrast, schistocytes in the non-TMA group were observed in conjunction with other moderate RBC abnormalities including crenated cells, poikilocytes, sickle cells, target cells, and nucleated RBCs in 46 cases (64.79%).

**Figure 1:**
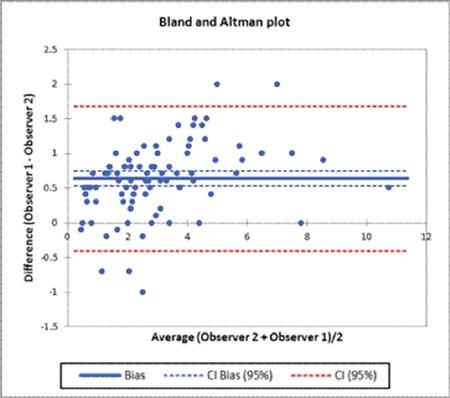
Graphical representation of the comparison of schistocyte percentages between the morphologists.

**Figure 2:**
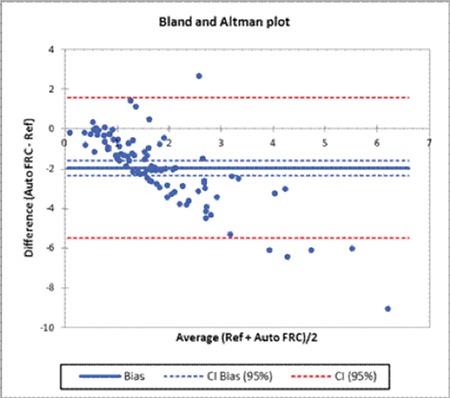
Graphical representation of the comparison of schistocyte percentages between the morphologists and the analyzer.

In the observer bias part of the study, the correlation coefficient between the two morphologists was 0.63 [confidence interval (CI): 0.52-0.75] (Figure 1). The mean schistocyte percentage of the morphologists was 2.3±1.86%. This was significantly higher than the FRC percentage of 1.05±1.33% (p<0.0001). The ICCC between the average of the morphologists and the FRC percentage was -1.97 (CI: -1.60 to -2.34) (Figure 2). Patients with TMA represented the majority of patients. The ADVIA underestimated the schistocyte count in patients with TMA. An overestimation of the schistocyte count was also noted in particular in the hemoglobinopathy group. This was owing to the presence of other moderate RBC abnormalities.

## DISCUSSION

The ICSH recommendations for the laboratory measurement of schistocytes were implemented at the CMJAH laboratory. The CMJAH is the second largest university hospital in Africa that offers specialist medical and surgical treatment including hematology and oncology. In this study, the ICSH recommendations for schistocyte identification and enumeration in 146 referred PBSs with schistocytes were evaluated.

In South Africa, there is a high incidence of TTP secondary to human immunodeficiency virus [9]. If the diagnosis is delayed, its clinical course can be rapidly fatal. According to the ICSH recommendations, the presence of ≥1% schistocytes on a PBS in the absence of other moderate RBC changes is a clinically significant criterion for the diagnosis of a TMA [3]. In this study, the mean schistocyte percentage in the TMA group (n=76) was 3.44±1.84%. This included 68 patients with a diagnosis of TTP. Studies have demonstrated that PBSs with the diagnosis of TTP present higher schistocyte counts when compared with other TMAs [2].

Schistocytes of ≥1% were, however, also observed in other nonfatal conditions. The mean schistocyte percentages of PBSs with the diagnosis of renal failure (n=10) or hemoglobinopathy (n=15) and in neonates (preterm, n=7; term, n=4) were 1.1±0.55%, 1.2±0.78%, and 1.55±0.8%, respectively. However, in the majority of the aforementioned conditions, schistocytes were observed in conjunction with additional moderate RBC abnormalities. This is consistent with the findings of Huh et al. [1]. Schistocytes in neonates are not pathological. A higher percentage is usually found in preterm neonates owing to liver immaturity. However, in this study, the term neonates presented with concomitant conditions that resulted in a slightly higher percentage than previously reported [1].

The diagnosis of TTP is based on clinical history, examination, and PBS review. However, according to the revised diagnostic criteria, the diagnosis of TTP should be considered in the presence of thrombocytopenia and microangiopathic hemolytic anemia alone [10]. In this study, the platelet count was significantly lower in the TMA group (p<0.001). The RDW was significantly higher in the TMA group whereas there was no difference for LDH. In this study, LDH isoenzyme fractions were not analyzed. Cohen et al. demonstrated that only LDH isoenzyme one and two fractions, the principal isoenzymes in RBCs, are significant markers of intravascular hemolysis [11]. In the TMA group the mean RDW was 22.34±4.29 (p<0.0001). Several authors have reported that RDW, a measure of anisocytosis, is an excellent screening tool for the presence of schistocytes, which is consistent with the findings of this study [12].

Although the presence of >1% schistocytes is a robust indicator of TMA, microscopic counts are variable among individual morphologists. In this study, the observer bias of two competent morphologists was analyzed for schistocyte enumeration according to the ICSH recommendations. Zini et al. proposed estimating the schistocyte percentage by counting a minimum of 1000 RBCs [3]. Although labor-intensive, in this study, it was necessary to count a minimum of 10,000 RBCs in order to estimate a precise count, as suggested by Rümke [13]. The authors were able to successfully reduce the observer bias by implementing the standardized procedures recommended by the ICSH. The correlation coefficient between the two morphologists was 0.63 (CI: 0.52-0.75). Nevertheless, subjectivity was found between the two morphologists in identifying ‘real’ schistocytes in the presence of anisopoikilocytosis. The ICSH defined specific morphology features of schistocytes: helmet cells; small, irregular, triangular, or crescent-shaped cells; pointed projections; and lack of central pallor [3]. The schistocyte counts between the morphologists showed discrepancies in the presence of acanthocytes, echinocytes, sickle cells, and crenated RBCs, as these RBC can be difficult to distinguish from schistocytes.

In contrast to microscopy, the automated FRC percentage is not dependent on the shape of the RBCs. The average microscopic schistocyte percentage was compared to the automated FRC percentage measured by ADVIA 2120 hematology analyzers. The mean schistocyte percentage of the morphologists was 2.3±1.86%. This was significantly higher than the automated FRC percentage of 1.05±1.33% (p<0.0001). The findings of this study differ from the findings reported by Lesesve et al. [6]. In that study by Lesesve et al., a range of normal and pathological specimens were included. In contrast, a limitation of this study is that only specimens with fragments were analyzed. The results of this study show that the ADVIA 2120 analyzer underestimated the schistocyte count in patients with TMA in the absence of other RBC abnormalities. An overestimation of the schistocyte count was also noted in the presence of anisopoikilocytosis. This is consistent with other reports that found that the ADVIA and Sysmex (Roche Diagnostics, Kobe, Japan) analyzers underestimated the schistocyte count after a threshold of 1.5% [6,7,14]. However, conditions such as hemoglobinopathies and renal failure represented a small percentage of the study population. Other studies also reported platelet interference in samples after platelet transfusions as another cause for overestimation of the automated FRC [14]. The automated FRC percentage requires further evaluation as a screening test.

## CONCLUSION

In conclusion, this study confirms that observer bias can be decreased by implementing the standardized procedures recommended by the ICSH. However, estimation of schistocytes by the ADVIA 2120 automated analyzer requires further evaluation as a routine diagnostic tool. A higher threshold for schistocytes in TTP should be considered in order to distinguish this hematological emergency from other conditions associated with ≥1% schistocytes.

## Figures and Tables

**Table 1:**
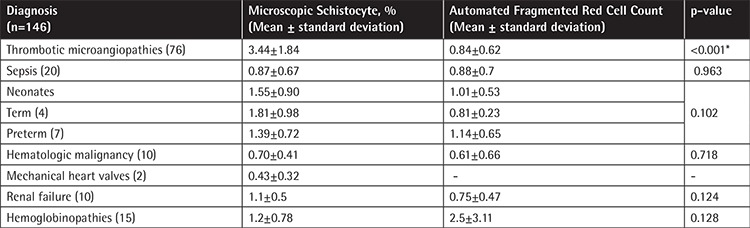
Microscopic and automated schistocyte percentages for each specific diagnosis.

**Table 2:**
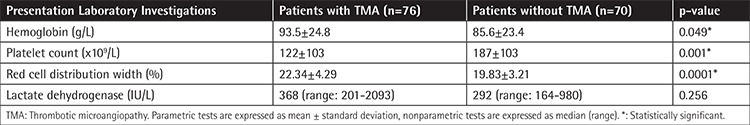
Presentation laboratory investigations (n=146).
